# Drug abuse results in metabolic and epigenetic changes in body which may contribute to disease risk: Role of L-carnitine and nutrients

**DOI:** 10.3389/adar.2023.10901

**Published:** 2023-01-18

**Authors:** Mohamed Ashraf Virmani

**Affiliations:** Medical and Scientific Affairs, Alfasigma BV, Utrecht, Netherlands

**Keywords:** metabolic, epigenetic, co-factors, L-carnitine, micronutrients, mitochondria, dopamine

## Introduction—Drug abuse and health risk

**FIGURE 1:**
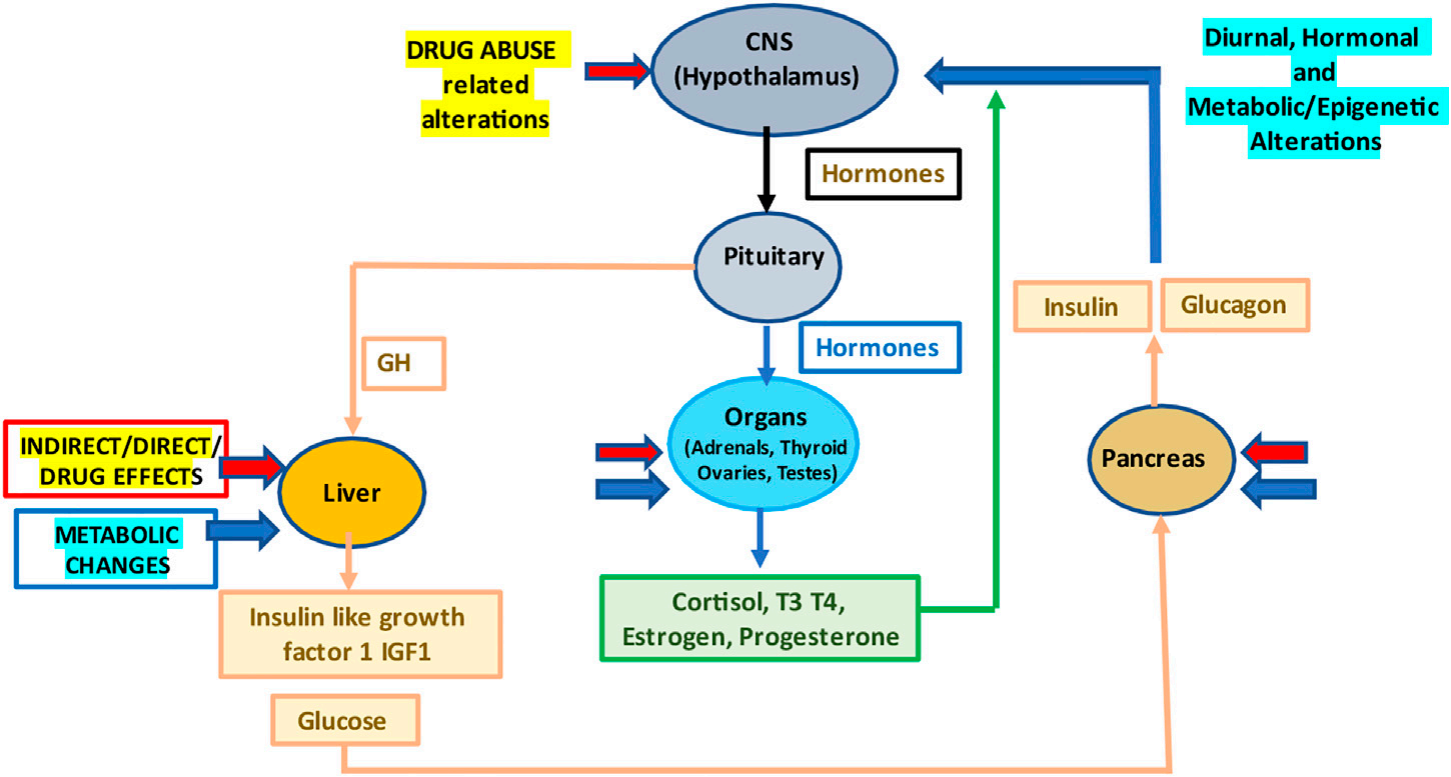
Drug abuse and the resulting diurnal, hormonal and metabolic/epigenetic changes in the body. Abbreviations: CNS, central nervous system; GH, growth hormone; T3, triiodothyronine; and T4, thyroxine.

Drug abuse is associated with significant health risk (1, 2). Studies are also showing that there is an important connection between circadian rhythms, metabolism and addiction (3–5). Study analyzing the liver metabolome of mice deficient in the expression of the dopamine D2 receptor (D2R) in striatal medium spiny neurons, found profound changes in the liver circadian metabolome compared to control mice (3). Further drugs that increase dopamine levels like cocaine disrupt circadian metabolic profiles in the liver. It is becoming evident that a strict communication exists between the CNS, and metabolism and this equilibrium can be altered by drug abuse (Figure 1). This loss of equilibrium in drug abusers may increase risk of metabolic dysfunctions which may result in worsening addiction and possible disease states such as those manifested as the metabolic syndrome.

## Drug abuse effects on metabolism and epigenetics

The metabolic syndrome is a collection of metabolic abnormalities, including hyperinsulinemia, hypertension, dyslipidaemia, and abdominal obesity, and may be triggered by initial discrepancies at the cellular level in critical metabolic pathways. These primary, small metabolic pathway disparities probably cascade with time leading in time to significant health problems. Some indications that drug abuse may increase the risk of the metabolic syndrome include the observation that drug-abusing patients have higher rates of diabetes complications. How drug abuse increases the risk to disease is being shown to be linked to changes in metabolism and epigenetics that are associated with the increased disease susceptibility. To counter these drug abuse related deleterious effects various studies, suggest that certain metabolic and antioxidant compounds like L-carnitine, thiamine (B1), co-enzyme Q10 etc., may be useful (6).

Drugs of abuse can be roughly categorised in different groups depending in part on their CNS activities, such as stimulants, amphetamines, hallucinogens, narcotics, inhalants, anaesthetics, anabolic steroids, and antipsychotics/antidepressants etc., (6). Each category and indeed each type of substance of abuse will affect metabolism differently depending on its unique chemical structure as well as the levels absorbed, processed and metabolised within the body. Furthermore, the effects on the metabolism within the body are manifested at various levels.

• **Cell functions** such as neurotransmission, release of hormones and inflammatory factors (cytokines, extracellular vesicles).
• **Intra-cellular functions** such as mitochondrial and endoplasmic reticulum (ER) activity.
• **Other body functions** such as temperature, intestinal absorption of nutrients, intestinal biota, function of vascular system.

Therefore, each drug of abuse affects overall metabolism in a complex way and the metabolic changes can eventually contribute to damage to cells and organs that manifest as tissue damage and eventually to manifestation of increased ageing rate and diseases (6, 7). Thus, for example, excess alcohol consumption leads mitochondrial dysfunction and inhibited lipid metabolism and reduced insulin sensitivity, eventually causing alcoholic fatty liver disease (8).

## Is it possible to protect against/reverse the metabolic and epigenetic changes due to drug abuse?

Every particular type of drug or substance of abuse has its own unique toxicity profile. Thus, amphetamines are known to affect the cardiovascular and neurological systems thereby exacerbating the risk factors for the metabolic syndrome. Methamphetamine users suffer cognitive deficits and abnormal metabolic activity, which affect nutritional status. The abuse of alcohol causes steatosis/liver injury as well as malnutrition leading to deficiencies in vitamin (B1, B2, B6, B12, C, K, A, and D) and minerals (selenium, zinc, magnesium, iron, and phosphorus) that significantly affect cellular metabolism as well as the epigenetic profile. The effects of these metabolic factors and their metabolites can impact gene expression by binding to transcription factors as well as by modifying chromatin structure and function (9–12).

In the common and chronic alcohol use disorder (AUD) the multiple exposure to ethanol produces an overall reduced anxiety which may be linked to opening of chromatin by increased histone acetylation, increased cAMP-response element binding protein (CREB) levels, and histone deacetylase (HDAC) inhibition (11, 12). Acute ethanol exposure was found to increase H3 and H4 histone acetylation as well as increase CREB and CBP in the central nucleus of the amygdala and to decrease HDAC activity in the amygdala (13). Since many organ systems are affected by alcohol, a cross-tissue and cross-phenotypic analysis, showed a differentially methylated region in the proprotein convertase subtilisin/kexin 9 (PCSK9) gene (11). This PCSK9 hypomethylation was conserved across tissues and positively correlated with expression.

Similarly, in the heroin abuse disorder methylation levels of CpG sites HTR1B_07 and HTR1B_26 and the promoter region of the HTR1B gene were hypermethylated in heroin abusers compared to healthy controls (14).

**FIGURE 2:**
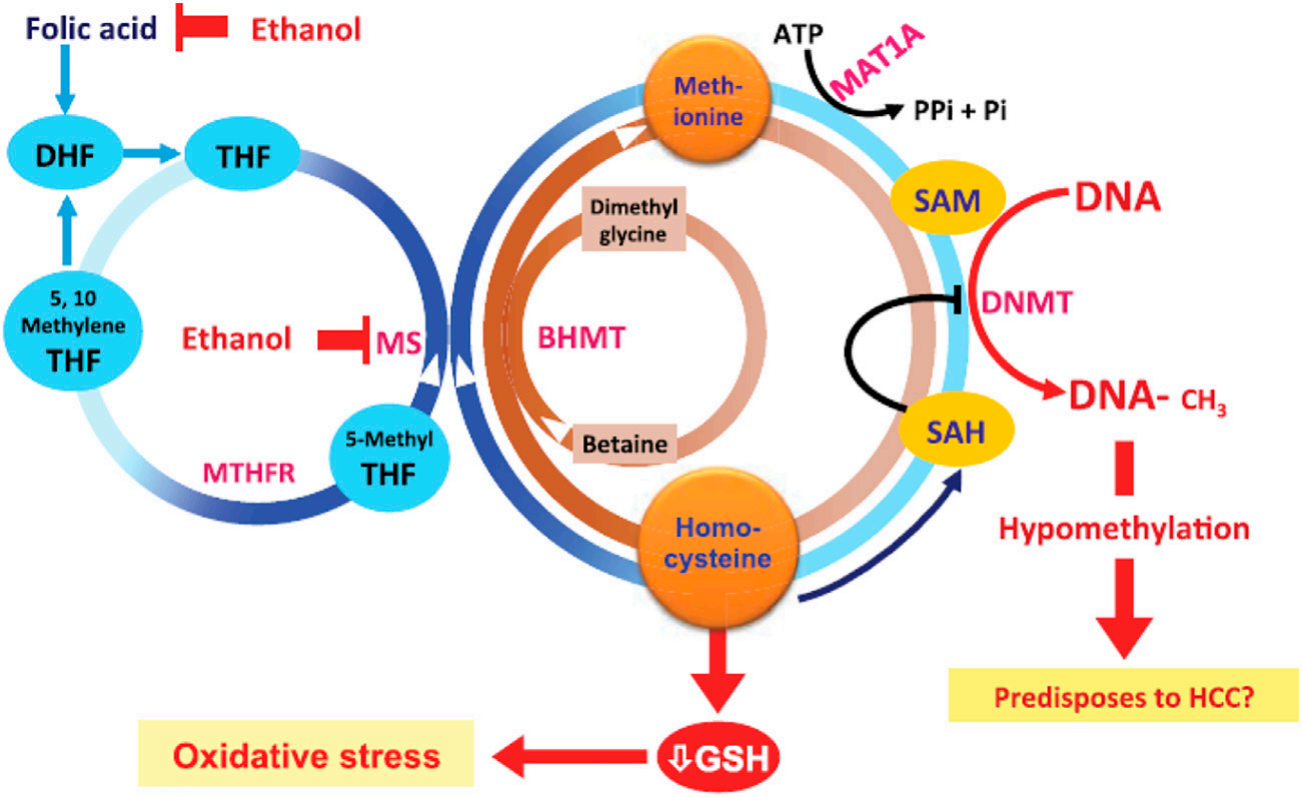
Effects of alcohol on metabolism and gene regulation mechanisms [from Ref (7)]. The effects of ethanol are *via* effects on homocysteine/methionine metabolism as well as DNA methylation. Abbreviations: ATP, adenosine triphosphate; BHMT, betaine homocysteine methyltransferases; DNMTs, DNA methyltransferases; GSH, glutathione; HCC, hepatocellular carcinoma; MAT, methionine adenosyltransferase; MTHFR, methylene tetrahydrofolate reductase; 5-methyl THF, 5-methyl tetrahydrofolate; MS, methionine synthase; Pi, inorganic phosphate; SAH, S-adenosylhomocysteine; SAM, S-adenosylmethionine.

**FIGURE 3:**
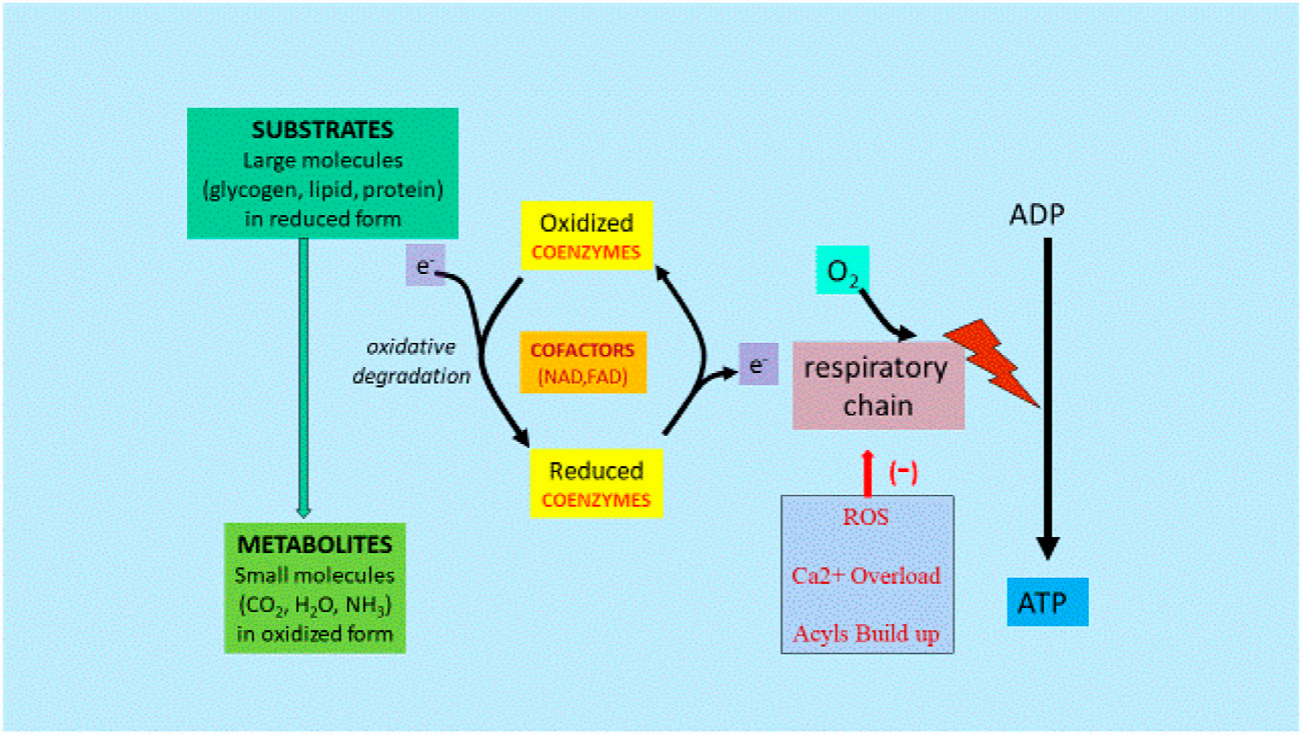
Metabolism and Gene Regulation Mechanisms [From Ref (4)]. Abbreviations: ADP, adenosine diphosphate; ATP, adenosine triphosphate; CO_2_, carbon dioxide; H_2_O, water; NH_3_, ammonia; Ca^2+^, calcium; e, electron; FAD, flavin adenine dinucleotide; NAD, nicotinamide adenine dinucleotide; ROS, reactive oxygen species.

The enzymes involved in epigenetic modifications of the DNA as well as the histone methylation/acetylation are reliant on specific micronutrient cofactors (Figure 3) and their activity can be directly impacted by the levels of these cofactors like nicotinamide adenine dinucleotide (NAD), flavin adenine dinucleotide (FAD) as well as metabolic compounds like L-carnitine, Q10, etc. Chronic alcohol consumption has also been reported to leads to significant reductions in micronutrient especially thiamine and has been reported to contribute to DNA hypomethylation (Figure 2). Thiamine deficiency in causes neurological injury leading to the Wernicke/Korsakoff syndrome (15).

## Future considerations

Studies are showing that drug abuse affects CNS as well as the diurnal rhythms of the whole body. These diurnal rhythm changes are also evident in at the level of cellular and organ metabolism (3). Drug abuse thus causes changes in gene expression and permanent epigenetic pattern changes which are related to the addiction and toxicity associated with long-term drug abuse. The metabolic and genetic/epigenetic dysfunctions over time in drug abuse may be countered or reduced by the use of micronutrients and metabolic cofactors, as well as by strategies utilising caloric restriction and preconditioning strategies. Exercise as well as environmental enrichment may be other strategies that impact on health of drug abusers (16). This may open ways to modulate the protective cellular pathways in a positive manner and reduce the impact of drug abuse.

Drug abuse related changes in metabolism would also have negative effects on various body systems due to increased cell damage, increased excitotoxicity, reduced energy production, and lowered antioxidant potential in cells. Partly this could also be due to mitochondrial dysfunction. This could be limited by adequate nutrient substrates and nutrient cofactors, especially L-carnitine, which play an important role in the mitochondrial function and metabolic flexibility (Figure 3).

Energy from starting substrates such as glycogen, lipids, and proteins are gradually extracted by the complex series of enzymes in the cellular cytoplasm and mitochondria, resulting in metabolites, mainly CO_2_ and H_2_O. Any dysfunction in this chain of events caused by lack of cofactors or mitochondrial dysfunction would lead to reduced ATP formation and increased ROS, Ca^2+^ and long chain fatty acid acyls and acylcarnitine buildup.

Further studies are needed to examine drug abuse related changes in gene/epigenetic expression and metabolism and to develop effective strategies to limit and reverse these changes. Indeed, certain metabolic compounds positively modulate the gene expression and associated protective cellular pathways and may contribute to limit drug abuse-related alterations and toxicity.
